# Modeling luminal breast cancer heterogeneity: combination therapy to suppress a hormone receptor-negative, cytokeratin 5-positive subpopulation in luminal disease

**DOI:** 10.1186/s13058-014-0418-6

**Published:** 2014-08-13

**Authors:** Aaron J Knox, Allison L Scaling, Mauricio P Pinto, Brian S Bliesner, James M Haughian, Hany A Abdel-Hafiz, Kathryn B Horwitz

**Affiliations:** 1 Department of Medicine, Division of Endocrinology, Metabolism and Diabetes, University of Colorado Anschutz Medical Campus, 12801 E. 17th Ave, Aurora, 80045 CO USA; 2 Departments of Pathology, University of Colorado Anschutz Medical Campus, Aurora, 80045 CO USA; 3 Center for Integrative Medicine and Innovative Science (CIMIS), Universidad Andrés Bello, School of Medicine, Santiago, Chile; 4 School of Biological Sciences, University of Northern Colorado, Greeley, 80639 CO USA

## Abstract

**Introduction:**

Many Luminal breast cancers are heterogeneous, containing substantial numbers of estrogen (ER) and progesterone (PR) receptor-negative cells among the ER+ PR+ ones. One such subpopulation we call “Luminobasal” is ER-, PR- and cytokeratin 5 (CK5)-positive. It is not targeted for treatment.

**Methods:**

To address the relationships between ER+PR+CK5– and ER–PR–CK5+ cells in Luminal cancers and tightly control their ratios we generated isogenic pure Luminal (pLUM) and pure Luminobasal (pLB) cells from the same parental Luminal human breast cancer cell line. We used high-throughput screening to identify pLB-specific drugs and examined their efficacy alone and in combination with hormone therapy in mixed-cell tumor models.

**Results:**

We show that pLUM and MCF7 cells suppress proliferation of pLB cells in mixed-cell 3D colonies *in vitro* and that pLUM cells suppress growth of pLB cells in mixed-cell xenografts *in vivo*. High-throughput screening of 89 FDA-approved oncology drugs shows that pLB cells are sensitive to monotherapy with the epidermal growth factor receptor (EGFR) inhibitors gefitinib and erlotinib. By exploiting mixed-cell 3D colonies and mixed-cell solid mouse tumors models we demonstrate that combination therapy with gefitinib plus the anti-estrogen fulvestrant constitutes a robust treatment strategy.

**Conclusions:**

We propose that response to combination endocrine/EGFR inhibitor therapies in heterogeneous Luminal cancers may improve long-term survival in patients whose primary tumors have been preselected for appropriate biomarkers, including ER, PR, CK5 and EGFR.

**Electronic supplementary material:**

The online version of this article (doi:10.1186/s13058-014-0418-6) contains supplementary material, which is available to authorized users.

## Introduction

Approximately 75% of breast cancers are luminal. They express estrogen receptors (ER) and/or progesterone receptors (PR) [1], tend to be hormone-dependent and are usually responsive to ER-targeted therapies [2]. Recently a review of immunohistochemical (IHC) ER and PR assays concluded that luminal cancers should be candidates for endocrine therapies if at least 1% of malignant cells are immunoreactive [3]. We asked: in such cases what are the other 99%, presumably receptor-negative, malignant cells? Indeed, the same question applies to less extreme tumors. The ER frequency distribution in 825 sequential breast cancers over a 2-year period [4] shows that although 81% of tumors are ER-positive (ER+), 30% to 80% of their cells are ER-negative (ER–). Analyses of 1,235 breast cancers [5] show PR distribution to be even more varied, with approximately 50% of PR+ tumors containing a significant proportion of PR– cells. Thus, most luminal tumors exhibit intratumoral heterogeneity containing substantial numbers of ER– and PR– cells among the ER+ and/or PR+ ones.

Intertumoral heterogeneity is well-known with breast cancer subtypes classified based on clinical and histopathological features, or gene expression profiles [6]. Major subtypes defined by gene profiling include luminal, human epidermal growth factor receptor-2-positive (HER2+) and basal-like. However, routine gene profiling is limited [7], and as all transcripts are pooled, the assay does not lend itself to analysis of intratumoral heterogeneity. Instead, five protein markers - ER, PR, HER2, Cytokeratin 5 (CK5) and epidermal growth factor receptor-1 (EGFR) - can serve as surrogates to classify breast cancers into subtypes analogous to those defined by gene profiling. Using these markers, IHC analysis of 10,159 invasive breast cancers collected from 12 studies [8] showed that in addition to 77% of tumors classified as luminal based on ER and/or PR positivity, approximately 6% are non-luminal but overexpress HER2 protein or its amplified gene [9], and approximately 16% are basal-like or triple-negative (TN) because they lack ER, PR and HER2. TN tumors were further subclassified into approximately 9% CK5+ and/or EGFR+ and approximately 7% were negative for all five markers. Clearly, such detailed intertumoral classification is important because each subtype responds differently to endocrine-, immuno- or chemotherapies, and each has a different long-term fate, with basal-like tumors generally characterized by heightened aggressiveness compared to luminal or HER2+ tumors.

Theories on the origins of intra- and intertumoral heterogeneity [10] include: rise of subclones from precursors that subsequently accrue genetic changes; stochastic gene expression patterns generating random signaling pulses; or cellular reprogramming by semi-stable imprinting of epigenetic tags [11]. The evolution and population frequency of modified subclones would then be regulated by the age and diagnostic stage of the tumor, differential growth rates, microenvironmental signals, cell-cell interaction dynamics, therapeutic interventions, and the like. There is also speculation based in part on the tissue organization of normal breast epithelium into luminal and basal elements, that the origins of intertumoral heterogeneity might be traced to normal stem cells that underlie these structures. If so, perhaps as also hinted at by expression profiling, one might speculate that the two major breast cancer subtypes - luminal and basal-like - should have negligible genomic kinship.

We find however, that the distinctions between intra- and intertumoral heterogeneity are not clear-cut. Recently we analyzed a series of luminal breast cancers using two markers - luminal PR and basal CK5 [12]. Four theoretical cell populations are possible and all were observed: PR+CK5– cells (luminal); PR–CK5+ cells (we dubbed luminobasal); cells lacking both markers (double-negative); and rare PR+CK5+ cells (double-positive). We also observed that more than 50% of luminal cancers contain luminobasal cells; that neoadjuvant endocrine therapies increase luminobasal-cell number in both hormone-responsive and hormone-resistant patients; and that in experimental models luminobasal-cell expansion can be blocked by inhibition of Notch signaling [13]. In retrospect, resistance of luminobasal cells to endocrine therapies is not surprising as they lack ER and PR and cluster with basal-like tumors. These facts suggested to us that presence of luminobasal cells in luminal disease might be dangerous and they needed to be targeted for treatment.

To define relationships between luminal and luminobasal cells and assess the therapeutic vulnerabilities of luminobasal cells, we have generated pure luminal (pLUM; ER+PR+CK5–) and pure luminobasal (pLB; ER–PR–CK5+) cell lines derived from solid T47Dco tumor xenografts. We characterize them, describe their biological properties when pure or mixed in three-dimensional colonies or as solid tumor xenografts, and use high-throughput screening to demonstrate that the pLB subpopulation is sensitive to EGFR inhibitors (EGFRi). We demonstrate that simultaneously targeting pLUM and pLB in mixed-cell tumors *in vivo* and three-dimensional colonies *in vitro* with a combination of the anti-ER fulvestrant plus the EGFRi gefitinib may constitute a robust treatment strategy for heterogeneous primary luminal disease expressing the appropriate biomarkers.

## Methods

### Cell lines

MCF-7 human breast cancer cells were from Sam Brooks (Michigan Cancer Foundation, Detroit); T47D cells were from Iafa Keydar (Tel Aviv University, Israel); the T47Dco subline was described in Horwitz *et al.*[14]. All cell lines have been authenticated by STR analysis and are mycoplasma-free.

### Generation of pLUM and pLB

All animal studies were approved by the University of Colorado Institutional Animal Care and Use Committee (approval number 91212 (02) 1E). Briefly, wild-type T47Dco cells in Matrigel were injected into cleared mammary fat pads of pre-pubertal ovariectomized (ovx’d) NOD SCIDγ (NSG) mice (NCI, Frederick, MD, USA or Jackson Laboratories, Bar Harbor, ME, USA) and implanted with cellulose (E withdrawn, EWD), 2 μg 17β-estradiol (E), or E + 8 μg progesterone (E + P)-containing pellets [13]. Three-month old tumors were minced, rotated with collagenase, DNAse and hyaluronidase followed by red blood cell lysis. Cells were plated in phenol-red free DMEM (Gibco) containing 5% charcoal-stripped fetal bovine serum (FBS) in EWD or 1 nM E-supplemented conditions.

To generate pLUM, cells from an E-tumor were propagated *in vitro* for approximately 2 months in 1 nM E. Live cells were sorted by fluorescence-activated cell sorting (FACS) (Moflo XDP 100, Beckman Coulter, Indianapolis, IN, USA) using CLD3 and CD49f to separate luminal (CLD3+ CD49f–) from luminobasal (CLD3– CD49f+) cells. The CLD3+ CD49f– population was replated, cultured for approximately 2 months more in E and re-sorted twice to generate pure pLUM (CLD3+ CD49f–). They were maintained in E-containing medium and remained luminobasal-free. To generate pLB, cells from an E + P tumor were plated *in vitro* for approximately 2.5 months under EWD conditions. They were sorted by FACS and the CLD3– CD49f + subpopulation was re-cultured for approximately 2 months more under EWD conditions then re-sorted twice to yield pure pLB (CLD3– CD49f+). They were maintained in EWD media and remained luminal-free. Both cell lines were authenticated by STR and are mycoplasma-free. Maintenance of pLUM and pLB states is monitored by IHC for a series of marker proteins (Table 1). Aliquots have been stably tagged with ZsGreen (ZsG) fluor [15].

**Table 1:** **Characterization of pure luminobasal (pLB) and pure luminal (pLUM) cells**

	Immunohistochemistry		mRNA
Factors	pLB	pLUM	Fold pLB versus pLUM
CK5	+++	-	+ 7,336
Jag1	+	-	+ 809
Annexin A1	+++	+	+ 405
EGFR	++	-	+ 146
Slug (SNAI2)	+	-	+ 103
p63 (TP63)	++	-	+ 65
CD49f (Itga6)	+++	-	+ 23
p-Cadherin (CDH3)	++	-	+ 20
Notch-1	+	-	+ 8
CD44	++	-	+ 1.3
CK8/18	+	+++	- 3
FOXA1	-	++	- 5
GATA3	-	++	- 46
ER	-	++	- 70
Claudin-3	-	++	- 113
Muc1	-	++	- 118
PR	-	++	- 189

CK5, cytokeratin 5; EGFR, epidermal growth factor receptor; ER, estrogen receptor; PR, progesterone receptor.

### Expression profiling

Briefly [16], 72 h 1 nM E-treated pLUM; EWD pLB; and E plus 100 nM P-treated T47Dco cells were suspended (Accumax; Millipore, Billerica, MA, USA), fixed, permeabilized with RNAlater (Ambion Inc., Ambion, Foster City, CA, USA) and incubated with anti-CK5 labeled with Zenon Alexa Fluor 488 (Invitrogen, Grand Island, NY, USA; Z-25002). Stained cells were centrifuged and resuspended in RNAse-free NST buffer containing 4′,6-diamidino-2-phenylindole (DAPI), and CK5+ versus CK5– cells sorted by FACS (Beckman-Coulter XDP-100 MoFlo). Separated cells were collected, centrifuged, and resuspended, and RNA was extracted (PicoPure; Arcturus/Life Technologies, Carlsbad, CA, USA). RNA from triplicate sorts was profiled using Agilent 4 × 44 K chips. Labeling, hybridization and initial analyses were performed at MOGENE LC, St. Louis, MO, USA. All microarray data can be accessed in the Gene Expression Omnibus database [GSE55350; GEO].

### Mixed-cell xenografts

pLUM (5 × 10^5^), ZsG-pLB alone, or 5:1 mixtures of pLUM:ZsG-pLB in Matrigel were injected into cleared mammary fat pads of ovx’d NSG mice implanted with cellulose (EWD) or E pellets. Tumor growth was quantified weekly for 8 weeks. At necropsy, tumors were resected, fixed in 4% paraformaldehyde, paraffin-embedded and processed for IHC. Paraffin sections were stained with DAPI, tumor boundaries were defined, scanned for ZsG fluorescence (Nikon T1 Eclipse) and quantified (NIS-Elements software; Nikon, Melville, NY, USA). For combination therapy experiments, 5 × 10^5^ pLUM, pLB alone, or 1:1 pLUM:pLB mixtures in Matrigel were injected as described above. Control 6 wk-old tumors generated from 1:1 mixtures were treated for 10 days with vehicles, gefitinib (100 mg/kg daily in 1% Tween; oral gavage), Fulvestrant (50 mg/mouse on days 1 and 6 in 10% ethanol and sesame oil; subcutaneously), or both. Tumor volumes were quantified every 48 h.

### Mixed cells *in vitro*

pLB (5 × 10^4^) or pLUM alone, or as 5:1, 3:1, 1:1, 1:3 or 1:5 pLUM:pLB ratios, were plated in E-free, growth factor (GF)-reduced Matrigel and grown into three-dimensional colonies for 7 days [17]. Alternatively, pLUM, pLB and MCF7 cells alone or as 1:1 or 5:1 pLUM:pLB or MCF7:pLB ratios were established over 24 h then treated for 7 days with: a) vehicles; b) 100 nM fulvestrant; c) 1 μM gefitinib on days 6 and 7; or d) fulvestrant plus gefitinib on days 6 and 7. Colonies were incubated for 1 h with bromodeoxyuridine (BrdU; Sigma, St. Louis, MO, USA), and Matrigel blocks were sandwiched between HistoGel, paraffin-embedded and sectioned for IHC [17].

### Immunohistochemistry (IHC)

Serial 4-μm sections were stained by dual IHC and DAPI for relevant luminal, basal, luminobasal and proliferation markers. Antibodies and sources are listed in Additional file 1: Table S1. Sections were photographed (Nikon Eclipse E600 fluorescence microscope) and cell subpopulations quantified (Image Pro 4.5; Media Cybernetics, Rockville, MD, USA). A BrdU index (BrdU+ nuclei in CK5+ or CK5– cells versus DAPI+ nuclei) was calculated from five random 100× (three-dimensional colonies) or 400× (xenografts) fields/condition.

### Cell-conditioned media

Cell-free (C) or pLB, pLUM or MCF7 cells were cultured to 50% to 60% confluence in DMEM with 5% FBS, then washed and incubated for 48 h in phenol red-free DMEM and charcoal-stripped FBS. Conditioned media (CM) were purged of cellular elements by filtration (0.2 μM). Separately, cells were grown for 48 h as three-dimensional Matrigel colonies in multiwall chambers. Medium was discarded and colonies were re-incubated with C or CM for an additional 48 h. Unfixed colonies were photographed (Nikon Eclipse E600) and cluster diameters were quantified from 100× brightfield images (Nikon NIS Elements).

### High-throughput drug screening

Mixtures (1:1) of ZsG-pLB (10^4^) and untagged pLUM (10^4^) were cultured for 24 h in triplicate 96-well plates in EWD or 2 nM +E conditions, then treated for 48 h with 0.05% DMSO or 1 μM drug from the 89-drug NCI-DTP Approved Oncology library (Additional file 2: Table S2). Media were aspirated, cells were fixed and immunostained with anti-CLD3, and counterstained with DAPI. Total (blue), pLUM (red) or pLB (green) subpopulations were imaged and quantified at 20×. Nine fields/well were captured (Additional file 3: Figure S1) averaging 8,000 cells/field in controls.

### Statistical analysis

Data were quantified as the mean ± standard error (SEM) of three or more separate experiments, and analyzed with Prism 6.0 (Graphpad Software, Lo Jolla, CA, USA) using one way analysis of variance (ANOVA) or Student’s *t*-test. Differences with *P* ≤0.05 were considered to be significant.

## Results

### Generation of pLUM and pLB cells

**Figure 1:**
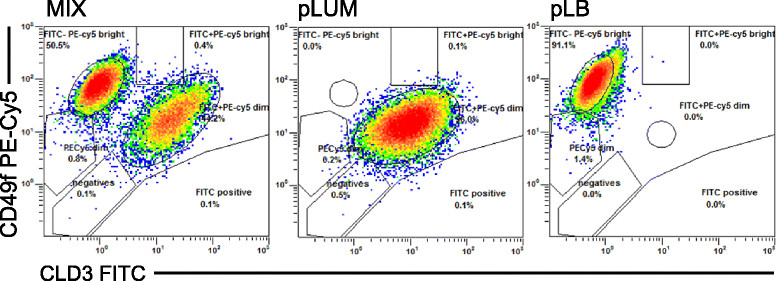
**Fluorescence-activated cell sorting (FACS) purification of pure luminal (pLUM) versus pure luminobasal (pLB) subpopulations.** Left panel: FACS of a mixed-cell T47Dco xenograft isolated from an estrogen (E) + progesterone (P) treated mouse, using CLD3- fluorescein isothiocyanate (FITC) (x-axis) and CD49f-PE-CY5 (y-axis), showing both cell populations. pLB (right panel) and pLUM (center panel) were separately collected and expanded in culture; cell lines were derived after re-sorting.

We recently isolated two cell lines from luminal T47Dco xenografts grown in ovx’d NSG mice: EWD8 consisting mainly of luminobasal ER–PR–CK5+ cells derived from a tumor in EWD mice; and E3 consisting mainly of luminal ER+PR+CK5– cells derived from a tumor in E-replenished mice [13]. Gene profiling, confirmed by IHC showed that CD49f expression was unique to EWD8 and CLD3 expression was unique to E3 [13]. Antibodies against these two proteins were used here for sequential dual FACS of another set of T47Dco mouse tumor-derived cells to generate two new, isogenic, pure cell lines: pLB are CLD3– CD49f+ and ER–PR–CK5+; pLUM are CLD3+ CD49f– and ER+PR+CK5– (Figure 1). Despite originating from the same parental cells each line exhibits a distinct gene signature (Additional file 4: Figure S2). pLB cells are propagated *in vitro* under EWD conditions; pLUM cells are propagated under E-replete conditions. Both have been tagged with ZsGreen [18].

To confirm markers unique to pLB or pLUM, expression of a 17-protein subset selected from the luminobasal gene signature [13] was assessed by IHC (Table 1). Proteins that marked pLB but not pLUM include CK5, Jag1, AnnexinA1, EGFR, Slug, p63 and CD49f; proteins that marked pLUM but not pLB include ER, PR, MUC1, CLD3, GATA3 and FOXA1. This confirms gene profiling transcript data (Table 1), the luminal assignment of pLUM, and the luminobasal assignment of pLB, which cluster with basal cells and tumors [13] but retain luminal markers like CK8/18. Like their parental cells, neither pLUM nor pLB express HER2 protein (not shown).

### Mixed-cell xenografts: pLUM cells suppress neighboring pLB cells

**Figure 2:**
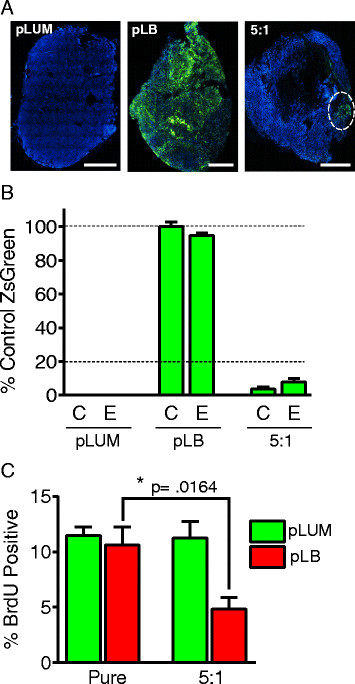
**Pure luminal (pLUM) suppress proliferation of neighboring pure luminobasal (pLB) in mixed-cell xenografted solid tumors. (A)** Whole-tumor cross-sections of untagged pLUM, ZsGreen (ZsG)-pLB and 5:1 pLUM:pLB xenografts grown in ovariectomized mice without hormones. Tumor sections counterstained with 4′,6-diamidino-2-phenylindole (DAPI) (blue). Scale bars equal 2000 μM. **(B)** Quantitation of ZsG fluorescence in pLUM, pLB and 5:1 pLUM:ZsG-pLB tumors relative to fluorescence of pure ZsG-pLB tumors under control (C) or estrogen (E) conditions. **(C)** Proliferation rates quantified by bromodeoxyuridine (BrdU) incorporation of pLUM and ZsG-pLB subpopulations in pure and mixed-cell tumor xenografts. Percent BrdU + cells in CK5– and CK5+ cells were quantified for ≥5 independent tumors; *P ≤0.05.

Most luminal tumors are heterogeneous, composed of cell mixtures including ER–PR–CK5+ luminobasal cells [12]. Because in TN disease luminobasal-like cells are highly aggressive we expected that in mixed luminal tumors the pLB subpopulation would eventually become dominant. However, in both luminal disease and *in vitro* models, ER–PR–CK5+ cells are indolent; proliferating more slowly than their ER+PR+CK5– neighbors [12]. To show if pLUM cells suppress nearby pLB cells the pure cell lines were used to control the ratio of each subpopulation in mixed-cell xenografts. Figure 2 shows data for 8-week-old tumors grown from pLUM, ZsG-pLB or a 5:1 pLUM:ZsG-pLB mixture in ovx’d mice without (C) or with E supplementation. The pure cell lines yield relatively pure tumors (green pLB or untagged pLUM) of each cell type (Figure 2A). However regardless of the hormonal state (Figure 2A, B) in 5:1 mixed pLUM:ZsG-pLB implants, the number of green pLB cells was significantly (*P* = 0.0164) suppressed. Despite starting at 20% of the population, only 4% to 8% of cells were pLB at necropsy (Figure 2B). Mixed-cell tumors tended to enlarge rapidly but had extensive necrotic centers and the rare pLB cells aggregated to tumor fringes (Figure 2A). Evidence for pLB suppression by pLUM cells was confirmed by BrdU quantitation (Figure 2C) showing that even in the absence of E (conditions in which luminal cells are unstimulated) pLUM cells suppress pLB proliferation.

### Mixed cells *in vitro*

**Figure 3:**
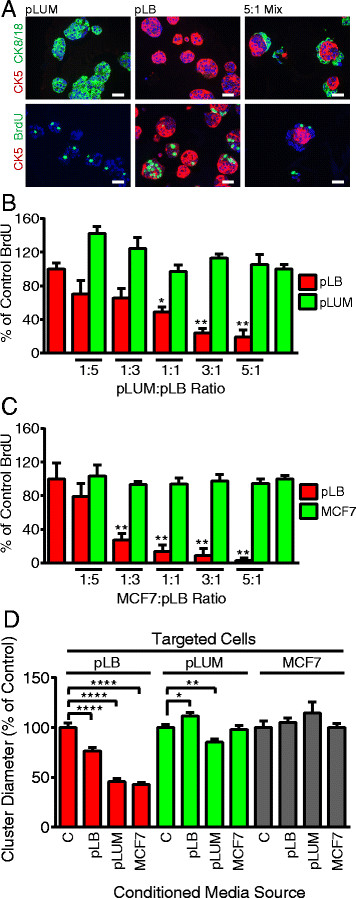
**Pure luminobasal (pLB) proliferation is suppressed by pure luminal (pLUM) cells via a diffusible factor.** pLUM, pLB and ratios of pLUM:pLB cell mixtures were seeded onto solidified growth factor (GF)-reduced Matrigel. Three-dimensional colonies were cultured for 7 days in hormone-free conditions. Bromodeoxyuridine (BrdU) was added, Matrigel blocks were sandwiched between HistoGel, paraffin-embedded and sectioned for immunohistochemistry (IHC). **(A)** Sections dual stained for CK5 (red) and CK8/18 or BrdU (green), counterstained with 4',6-diamidino-2-phenylindole (DAPI) (blue). Scale bars equal 20 μM. **(B)** Quantitation of BrdU incorporation into pLB or pLUM cells in pure cells (set at 100%) or mixed-cell colonies. **(C)** Same as A and B, but MCF-7 were substituted for pLUM. **(D)** Media-conditioned (CM) by no cells (control), or by pLB, pLUM or MCF7 cells growing in 2D on plastic, were collected. Separately, pLB, pLUM or MCF-7 cells were grown as three-dimensional Matrigel colonies in regular media for 48 h, then switched to the CM media shown for 48 h. Colonies were photographed and their diameters in CM were compared to colony diameters in control media; *P ≤0.05; **P ≤0.01; ***P ≤0.001; ****P ≤0.0001.

To address mechanisms for pLB suppression by pLUM, single-cell suspensions of pLUM and pLB cells either alone or mixed in pLUM:pLB 5:1, 3:1, 1:1, 1:3 or 1:5 ratios were grown as three-dimensional colonies under EWD conditions [17]. Seven-day colonies were incubated with BrdU, sectioned and dual-stained for BrdU plus luminal CK8/18 or basal CK5 to mark each subpopulation (Figure 3). Note that despite having been introduced into Matrigel co-mingled, cells aggregate by type with mixed-cell colonies characteristically having a pLB core surrounded by pLUM (Figure 3A, top). In general, pLUM cells have a higher proliferation rate than pLB cells (Figure 3A bottom, B), and pLB proliferation progressively shrinks as the proportion of pLUM rises (Figure 3B). At 5:1 pLUM:pLB ratios proliferation of pLB is reduced by approximately 81% (*P* <0.0001) compared to pLB controls.

To demonstrate that suppression by pLUM of pLB is a general phenomenon, the three-dimensional mixed-cell colony assay was repeated substituting classic luminal ER+PR+ MCF-7 cells for pLUM (Figure 3C). MCF-7 cells are powerful suppressors, progressively reducing pLB proliferation with 97% (*P* <0.0001) pLB suppression at the 5:1 MCF-7:pLB excess.

As mixed cells segregate by subtype within colonies and tumors with minimal direct pLUM and pLB cell-cell contact, we speculated that pLUM suppress pLB via a diffusible factor. To test this, pLB, pLUM or MCF-7 cells growing as three-dimensional colonies were incubated with media previously conditioned (CM) by pLB, pLUM or MCF-7 cells grown on plastic. Compared to control cell-free medium, pLUM (Figure 3D) or MCF-7 CM (not shown) significantly reduced the size of pLB colonies by >50% (*P* <0.0001) whereas pLB CM had little effect on pLUM or MCF-7 (Figure 3D) colonies. This suggests that diffusible inhibitory factor(s) released by luminal cells suppress proliferation of nearby luminobasal cells.

### pLB cells are selectively targeted by EGFR inhibitors

**Figure 4:**
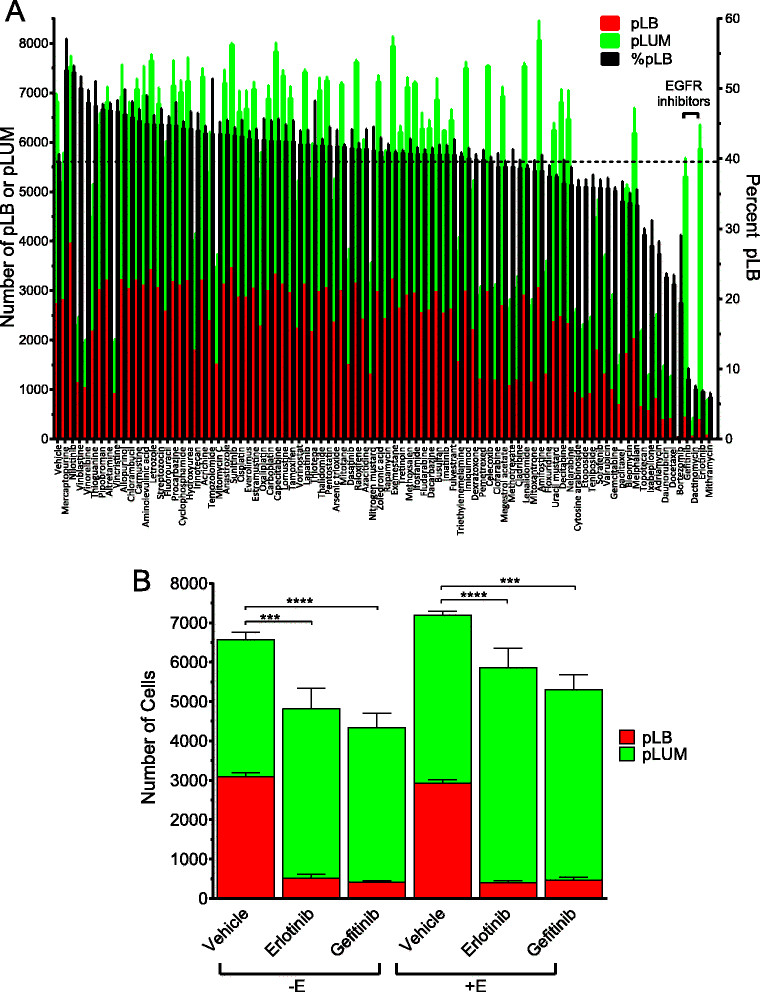
**High-throughput screening: targeting pure luminobasal (pLB) cells with drugs from an NCI-approved 89-drug oncology library. (A)** 1:1 mixtures of pLUM:ZsG-pLB were co-cultured in triplicate 96-well plates in 1 nM 17β-Estradiol (E; not shown, see panel **B**) or E-free (shown) conditions, then treated 48 h with a panel of 89 NCI-approved oncology drugs (1 μM) or 0.05% dimethyl sulfoxide (DMSO) controls. Media were aspirated, cells were fixed, immunostained for CLD3 (red) and counterstained with 4',6-diamidino-2-phenylindole (DAPI) (blue). Total (blue), pure luminal (pLUM) (red) or pLB (ZsG, green) subpopulations were imaged and quantified at 20×. There were 9 fields/well captured, averaging 8,000 cells/field in controls (Additional file 3: Figure S1A). Mean numbers of pLB (red bars) and pLUM (green bars) cells, and % pLB (black bars) are shown as the average of triplicates ± standard error of the mean (SEM). Erlotinib, *P* ≤0.0001, gefitinib, *P* = 0.0005. **(B)** Details of vehicle, erlotinib or gefitinib data from panel A in both +E and –E conditions; ***P ≤0.001; ****P ≤0.0001 for pLB cell numbers in gefitinib or erlotinib versus vehicle control.

In mixed-cell hormone-dependent tumors, luminobasal cells are intrinsically hormone- and chemotherapy-resistant [12],[13]. This scenario may be acceptable in untreated cancers containing few luminobasal cells. The danger arises when the number of luminobasal cells expands as ER-targeted therapies reduce luminal cell number [12]. We therefore suspect that luminobasal cells should be treated in primary tumors. To identify drugs that selectively do so, the 89-drug NCI-DTP Approved Oncology library (Additional file 2: Table S2) was screened. It contains hormone antagonists, chemotherapeutic agents, target-based kinase inhibitors and epigenetic/histone acetylation-modifying drugs in current clinical use. A high-throughput screen used fluorescent imaging to quantify ZsG-pLB cells and CLD3+ pLUM cells plated in 1:1 mixtures. Cells were pretreated for 24 h under EWD or +E conditions (Additional file 3: Figure S1), after which pLB comprised 45% of cells in EWD wells and 39% in +E wells. While EWD or +E were continued, the cells were exposed to drugs for 48 h. Figure 4A summarizes the number of pLB (red), pLUM (green) and % pLB (black) compared to vehicle controls under +E conditions. Extensive nonspecific cytotoxicity for both cell types was induced by chemotherapeutic agents such as vincristine, azacytidine, gemcitabine, mithramycin, et cetera. However, selective and targeted reduction of the pLB subpopulation (note the drop in % black pLB bars with conservation of green pLUM bars) was observed with the anti-EGFR small molecule inhibitors gefitinib and erlotinib (Figure 4A). Detailed analyses under both EWD and +E conditions (Figure 4B) showed that after 48 h gefitinib reduced the pLB subpopulation from 45% to 9% (EWD) (*P* ≤0.0001) and from 39% to 9% (+E) (*P* = 0.0005). Similarly, erlotinib reduced the pLB subpopulation to 10% (EWD) (*P* = 0.0001) and 7% (+E) (*P* ≤0.0001). Neither drug influences the pLUM subpopulation.

The reliance on EGFR signaling correlates with upregulation of EGFR in pLB cells [13] (Table 1; Additional file 5: Figure S3). Interestingly, during the 48 h treatment time the dual HER2/EGFR inhibitor lapatinib, currently in use for second-line therapy of herceptin-resistant ER+EGFR+HER2+ breast cancers, did not reduce the pLB subpopulation, consistent with the fact that HER2– tumors (like pLB) tend to be unresponsive to this drug [19]. Additionally, nilotinib, a *bcr-abl* inhibitor produced an unexpected and significant increase in the proportion of pLB cells, an effect that requires further study. Lastly, the pLUM subpopulation was unaffected by the endocrine therapeutic drugs present in the library. In our experience, in 2D conditions like those used here these require a longer treatment window (but see 3D, Additional file 6: Figure S4).

### Combination anti-ER/anti-EGFR therapy targets both populations in mixed-cell tumors

**Figure 5:**
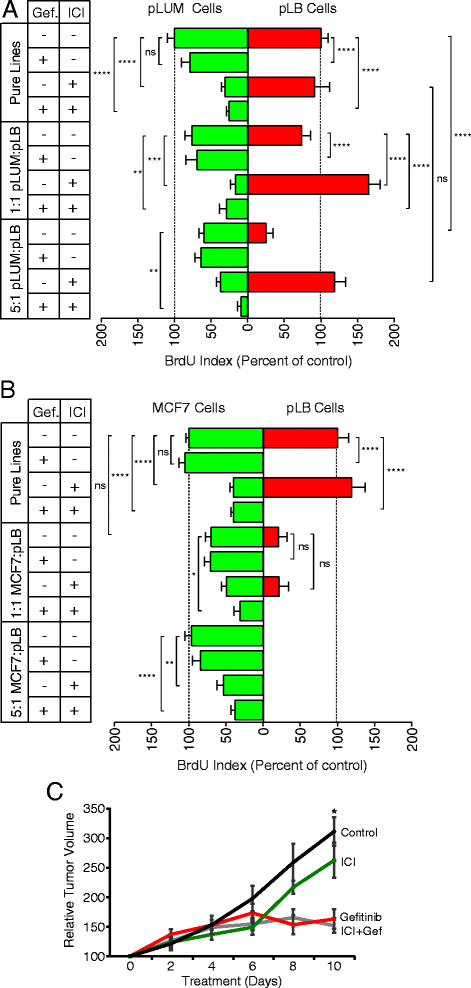
**Combined targeting of pure and mixed-cell pure luminal (pLUM) and pure luminobasal (pLB) colonies with fulvestrant/gefitinib. (A)** Pure pLUM, pLB or 1:1 and 5:1 pLUM:pLB mixtures were grown as three-dimensional Matrigel colonies in the absence of hormones for 48 h. Colonies were switched for 5 days to media containing no drug (days 1 to 5); 1 uM gefitinib (days 4 to 5); 100 nM ICI (days 1 to 5); or both (ICI days 1 to 5; gefitinib days 4 + 5). Colonies were treated with bromodeoxyuridine (BrdU), processed for immunohistochemistry (IHC) and a BrdU proliferation index was quantified. Data are average of triplicates ± standard error of the mean (SEM). *P ≤0.05; **P ≤0.01, ***P ≤0.001, ****P ≤0.0001, ns, not-significant. **(B)** Same as **A** except MCF-7 cells were substituted for pLUM. **(C)** 1:1 mix of pLUM:pLB cells was injected into cleared fat pads of ovariectomized non-obese diabetic/severe combined immunodeficient (NSG) mice and tumors were grown 6 weeks without hormones. Mice were treated 10 days with no drug (Control), fulvestrant (ICI), gefitinib or both. Tumor volumes are expressed as % volume on treatment day 0, for ≥4 tumors ± SEM at each time point.

Our goal is to proffer combination therapies that target luminal and luminobasal subpopulations in heterogeneous ER+PR+ disease. We tested gefitinib in combination with fulvestrant (ICI 182, 780; ICI) using 1:1 or 5:1 pLUM:ZsG-pLB mixtures in three-dimensional colonies (Figures 5, Additional file 6: Figure S4A) treated with vehicle, ICI (100 nM), gefitinib (1 μM) or ICI + gefitinib. BrdU was added prior to harvest, colonies were fixed, sectioned and stained for CLD3, CK5 and BrdU, photographed and a BrdU index/cell type was quantified. Gefitinib completely suppressed pLB proliferation with no effect on pLUM. ICI significantly reduced pLUM with no significant effect on pLB. Combining the drugs showed no influence of one on the other (Figure 5A). Drug effects in 1:1 and 5:1 pLUM:pLB colonies were highly effective: the combination decreased proliferation in 1:1 pLUM:pLB colonies by 88.5% (*P* <0.0001), and in 5:1 colonies by 95% (*P* <0.001). Additionally the cell heterogeneity exposed effects of one cell type on the other. For example, suppression of pLUM by ICI monotherapy paradoxically increased pLB proliferation in the absence of gefitinib by removing the pLUM-secreted inhibitor (Figure 3). We argue that this can be prevented in cell mixtures by combination therapies that target both cell types. In sum the data point to the value of targeting two cell subpopulations in a tumor, and provide an illustration that monotherapy targeting one subpopulation, can have the unintended consequence of allowing the other to expand.

To demonstrate that this outcome was not restricted to one cell line, we substituted MCF-7 cells for pLUM cells (Figure 5B). This shows again the marked suppressive effects of MCF-7 cells on proliferation of neighboring pLB cells even without treatment, the further proliferative suppression of pLB by gefitinib, and the efficacy of the combination therapy in reducing overall colony cell growth. Photographs of representative colonies stained for CK5 (red), BrdU (green) and total cells (blue) are shown for pLUM:pLB (Additional file 6: Figure S4A) and MCF-7:pLB (Additional file 6: Figure S4B). Note for example that gefitinib completely shuts down proliferation of pure pLB colonies (panel 5); or that only luminal cells in mixed colonies proliferate with combination therapies (panel 16).

We then tested a gefitinib/ICI combination in heterogeneous solid tumors *in vivo* (Figure 5C)*.* For this 5 × 10^5^ pLUM, pLB or a 1:1 pLUM:pLB mixture was injected into cleared mammary fat pads of ovx’d mice without hormone supplementation and grown into tumors for 6 weeks. Since pLUM cells suppress pLB cells (Figures 2 and 3) the 1:1 ratio was chosen so that sufficient numbers of pLB cells would remain in 6-week-old tumors to allow their quantitation despite further EGFRi suppression. Six-week tumor-bearing mice were administered vehicle controls, ICI (5 mg/mouse; days 6 + 10), gefitinib (100 mg/kg, once daily), or ICI + gefitinib for 10 days. Tumor sizes were measured every 48 h. Tumors continued to expand in controls during 10 days; they were modestly reduced by ICI (which requires longer treatment times) and significantly reduced by gefitinib alone (*P* = .0005) or the gefitinib + ICI combination (*P* = .0002). These data demonstrate that EGFRi can target LB cells even in the presence of an anti-estrogen.

## Discussion

### Hormone resistance

Treatments for luminal breast cancer exploit its ER-positivity and estrogen dependence by disrupting estrogen signaling, downregulating the ER or reducing E production. Patients with luminal disease respond well initially, but long-term survival declines steadily after 5 years and eventually falls below that of basal-like cancers [20],[21]. Mechanisms underlying luminal tumor recurrence are varied. Explanations include development of acquired resistance through genetic or epigenetic pathways that target ER signaling or upregulate growth factor escape, or pre-existing resistance due, for example, to mutant ER [22]. All likely apply to subsets of patients. However, directly or indirectly all assume that ER+ cells - either early or late in luminal tumor evolution - undergo the molecular changes linked to resistance. The present study focused on an alternative mechanism, namely that many primary luminal tumors are heterogeneous at diagnosis, containing ER– cells that lack the molecular machinery to respond to endocrine therapies. Although the origins of ER– cells in luminal disease remain under intense study [23], we argue that such cells cannot be ignored and should be treated along with their ER+ neighbors. Indeed we have shown in both clinical and experimental settings that targeting only the ER+ subpopulation has the unintended consequence of increasing the ER– subpopulation, perhaps spawning acquired heterogeneity. Our goal here has been to discover drugs for treatment of luminal cancers that would suppress the intrinsic or acquired ER–PR–CK5+ subpopulation when combined with standard endocrine therapies. For that we needed pure luminal-derived ER–PR–CK5+ cells for drug screening, that is, the pLB cells reported here (Figure 1).

### Luminal tumor-cell heterogeneity

Assessment of intratumoral heterogeneity requires cell-by-cell or microdissection methods that do not assume a tumor is a uniform mass of identical cells. This is in contrast to studies using bulk tumor DNA, RNA or protein for analysis of gene expression patterns, mutation spectra, copy number changes, et cetera. [24]. Although the latter clearly show that even within major breast cancer subtypes like luminal there are clinically important subsets that modify patient outcome and necessitate unique therapeutic approaches, they do not address intratumoral cell heterogeneity. Some studies have used laser capture microdissection to demonstrate intratumoral cytogenetic heterogeneity [25], or heterogeneity of *c-myc*, *cyclinD1*[26], HER2 [27] or ER [28] expression. We used ER/PR and CK5 as markers to show that significant cellular variability exists within and between luminal tumors [12] with ER–PR–CK5+ cells interspersed among the ER+PR+CK5– cells in more than 50% of cases. Clearly this presents significant diagnostic and therapeutic challenges, including the risk of flawed prognostic estimates and inadequate treatment based on reliance on a single, possibly unrepresentative, biopsy. Transcending these is the failure to treat subpopulations of tumor cells that are not identified by current assays [23],[29]-[31].

### Modeling luminal heterogeneity

Here we developed pure ER+PR+CK5– and ER–PR–CK5+ cell lines derived from the same parental luminal precursor that allow us to reconstruct intratumoral heterogeneity while tightly controlling the ratio of these two cell populations. Among other things, we needed to control luminobasal cell numbers because they are often sparse in untreated mixed-cell luminal cancers. Additionally, although the origins of intratumoral heterogeneity remain unclear, with both cancer stem cell and clonal evolution hypotheses proffered, in either scenario cell variability is posited to originate from a single cell [29]. This renders the progeny genetically identical. We sought to model this property as well. To our knowledge pLUM and pLB represent rare examples of natural isogenic cells exhibiting substantive phenotypic differences. To generate them, fixed CK5– and CK5+ cells sorted by FACS from the same tumor xenograft were profiled to discover genes that encode cell-surface proteins and discriminate between the two populations [13]. This allowed us to develop a novel FACS-based scheme using CLD3 and CD49f to separate the two cells from a parental-cell mixture in a sterile, live state (Figure 1). Successive purifications yielded highly pure cell lines that maintain pLUM or pLB phenotypes in culture and provide a stable resource for modeling and manipulating heterogeneity. As the cells share a common progenitor and exhibit little genetic divergence [13] this limits the influence of unique mutations on their biological behavior and response to drugs.

### Luminal cells suppress luminobasal cells

Clinical evidence [12], *in vitro* three-dimensional colonies (Figure 3) and *in vivo* solid tumor xenografts (Figure 2) demonstrate that luminal and luminobasal cells in a mixed-cell microenvironment behave differently than the same cells in pure states, suggesting that there is cross-talk between them. Heterogeneous mixed-cell xenografts are larger but more necrotic than their homogenous pure-cell counterparts (Figure 2). In mixed three-dimensional colonies, pLUM proliferation is stimulated by presence of pLB cells (Figure 3) or by LB-conditioned media (Figure 3D). The soluble factors involved are unknown but could include insulin-like growth factors known to be high in ER– cells, low or absent in ER+ cells, but induce ER and promote ER+ tumor growth [32],[33]. In contrast pLB cell proliferation is suppressed by neighboring pLUM or MCF7 cells (Figure 3), which also involves secreted factors (Figure 3D). The paracrine-acting inhibitory factors are also unknown but could include transforming growth factor (TGF)-β [34],[35]. The findings of LB suppression by LUM, plus our clinical data showing that luminobasal cells are upregulated in tumors of tamoxifen-treated patients [12] present a troubling scenario. Namely, that prior to antiestrogen therapy a minor intrinsic luminobasal subpopulation is suppressed by dominant neighboring luminal cells. However, as antiestrogens shrink inhibitory signals coming from the luminal population, the luminobasal population expands; this is an illustration of inadvertent therapy-induced enrichment [33],[36]. These findings underscore the impact that intratumoral cellular dynamics have on the success (or failure) of endocrine therapies in heterogeneous tumors, prompting us to seek drugs that directly target the ER–CK5+ subpopulation.

### Combination therapy targeting Luminal tumor heterogeneity

To find drugs that suppress the luminobasal subpopulation, co-cultured pLUM and pLB cells were arrayed and screened for pLB-specific drugs using a library of 89 FDA-approved and well-characterized oncology therapeutics. The two EGFRi in the library, gefitinib and erlotinib, were highly pLB-specific. This was in contrast, for example, to the EGFR/HER2 inhibitor lapatinib, which showed no selectivity (Figure 4 and Additional file 2: Table S2). Identification of EGFRi as luminobasal-specific agents led us to analyze a dual antiestrogen/EGFRi combination that simultaneously targets pLUM and pLB in mixed-cell three-dimensional colonies (Figure 5A, 5B) and xenografts (Figure 5C). We observed a strong response to brief gefitinib treatment, even in the presence of fulvestrant, demonstrating that the EGFRi is bioactive in combination with an antiestrogen.

Clinically, EGFRi monotherapy or antiestrogen/EGFRi combinations have yielded mixed results. In advanced luminal disease previously treated with antiestrogens, gefitinib had no detectable benefit, perhaps because the tumors contained few EGFR+ cells [37]. On the other hand, in metastatic disease, Cristofanilli *et al*. observed that gefitinib in conjunction with anastrozole prolonged progression-free survival [38]. With regard to therapy for early stage disease, addition of gefitinib to anastrozole failed to improve outcome in one study [39], while in another, gefitinib combined with tamoxifen was beneficial in previously untreated patients [40]. Clinical benefits of EGFR inhibition may stem from delaying acquisition of hormone resistance [38] or from blocking proliferative growth factor signaling in tumors with EGFR+ cells. In support of this, neoadjuvant treatment of primary tumors with gefitinib or gefitinib plus anastrozole in a postmenopausal, EGFR+ cohort reduced proliferation, EGFR activation and tumor size [41]. We propose that response to combination endocrine/EGFRi therapies in ER+ luminal cancers may improve long-term survival in patients whose tumors have been preselected [42] for presence of a luminobasal subpopulation based on ER/PR, CK5 and EGFR biomarker expression.

## Conclusions

Currently, the ER–PR–CK5+ cells of luminal breast cancers are not treated. They are malignant, indolent and antiestrogen-resistant; they expand in response to endocrine or chemotherapies [21]; at least a subset has tumor-initiating capacity [12],[13],[43]. We now show that in mixed-cell experimental models, the ER–PR–CK5+ population can be suppressed by EGFRi and that combination endocrine/EGFRi therapies may additively target ER+PR+CK5– and ER–PR–CK5+ subpopulations. Clinically, luminal breast cancers exhibiting the appropriate heterogeneity for treatment by this combination could be easily identified using ER/PR and CK5 or EGFR as biomarkers. As EGFRi are already approved for oncology use, the combination therapies we propose can be immediately translated to clinical trials.

## Authors’ information

Aaron J Knox and Allison L Scaling: first authors.

## Additional files

## Electronic supplementary material

Additional file 1: Table S1.: Antibodies used in study. (PDF 173 KB)

Additional file 2: Table S2.: 89 NCI-DTP Approved Oncology Library screen of luminobasal cells. (PDF 36 KB)

Additional file 3: Figure S1.: Luminal and luminobasal coculture assay for luminobasal-specific therapies. (PDF 2 MB)

Additional file 4: Figure S2.: Volcano plot of differentially expressed genes in pure luminal (pLUM) versus pure luminobasal (pLB) cells. (PDF 611 KB)

Additional file 5: Figure S3.: Epidermal growth factor receptor (EGFR) expression in luminal versus luminobasal subpopulations: fluorescence-activated cell sorting (FACS). (PDF 404 KB)

Additional file 6: Figure S4.: Combined targeting of luminal and luminobasal cells: Antiestrogen/Gefitinib treatment of mixed-cell tumor models. (PDF 6 MB)

Below are the links to the authors’ original submitted files for images.

Authors’ original file for figure 1

Authors’ original file for figure 2

Authors’ original file for figure 3

Authors’ original file for figure 4

Authors’ original file for figure 5

## Abbreviations

BrdU: bromodeoxyuridine
CK5: cytokeratin 5
CM: conditioned media
DAPI: 4′,6-diamidino-2-phenylindole
DMEM: Dulbecco’s modified Eagle’s medium
DMSO: dimethyl sulfoxide
E: estrogen
EGFR: epidermal growth factor receptor
EGFRi: epidermal growth factor receptor inhibitor
ER: estrogen receptor
EWD: estrogen withdrawn
FACS: fluorescence-activated cell sorting
FBS: fetal bovine serum
GF: growth factor
ICI: ICI 182 780, fulvestrant
HER2: human epidermal growth factor receptor-2
IHC: immunohistochemistry
NSG (NOD SCIDγ): non-obese diabetic/severe combined immunodeficient
ovx'd: ovariectomized
P: progesterone
pLB: pure luminobasal
pLUM: pure luminal
PR: progesterone receptor
STR: short tandem repeats
TN: triple negative
ZsG: ZsGreen
