# Supplementary material for: Modeling luminal breast cancer heterogeneity: combination therapy to suppress a hormone receptor-negative, cytokeratin 5-positive subpopulation in luminal disease
Source: Breast Cancer Res. 2014 Aug 13;16:418. doi: 10.1186/s13058-014-0418-6 (PMC4187339; doi:10.1186/s13058-014-0418-6)
Supplement: Supplementary file 1 — Additional file 1: Table S1.: Antibodies used in study. (PDF 173 KB) [file 13058_2014_418_MOESM1_ESM.pdf]

Additional File 1: Table S1. Antibodies

Antibody	Application	Dilution	Source
Annexin A1	IHC	1:100	Millipore
BrdU	IHC	1:50	Becton-Dickinson
CD44	IHC	1:200	Neomarkers/Thermo Sci
CD49f	IHC	1:100	Millipore
CD49f PE-Cy5	FC	–	BD Pharmingen
CK8/18	IHC	1:100	Leica Microsystems
CK5	IHC	1:200	Abcam
CK5	IHC, FC	1:100	Leica Microsystems
Claudin-3	IHC	1:200	Abcam
Claudin-3 FITC	FC	–	R&D Systems
EGFR	IHC	1:400	Millipore
EGFR-PE	FC	–	BioLegend
ER (Clone 1D5)	IHC	1:50	Dako
ERα (SP1)	IHC	1:100	Labvision
FOXA1	IHC	1:500	Abcam
GATA3	IHC	1:100	Santa Cruz Biotech
Jag1	IHC	1:100	Santa Cruz Biotech
Muc1	IHC	1:100	Santa Cruz Biotech
Notch-1	IHC	1:100	Cell Signaling
Notch-1 APC	FC	–	BioLegend
P-Cadherin	IHC	1:100	R&D Systems
PR (SP2)	IHC	1:100	LabVision
PR (Clone 1294)	IHC	1:500	Dako
P63	IHC	1:50	Epitomics
Slug	IHC	1:50	Cell Signaling
**Secondary Antibodies**			
Goat anti rabbit 488	IHC	1:200	Invitrogen
Goat anti-rabbit 555	IHC	1:200	Invitrogen
Goat anti-mouse 488	IHC	1:400	Invitrogen
Goat anti-mouse 555	IHC	1:200	Invitrogen

IHC: Immunohistochemistry, FC: Flow Cytometry
