# Supplementary material for: Modeling luminal breast cancer heterogeneity: combination therapy to suppress a hormone receptor-negative, cytokeratin 5-positive subpopulation in luminal disease
Source: Breast Cancer Res. 2014 Aug 13;16:418. doi: 10.1186/s13058-014-0418-6 (PMC4187339; doi:10.1186/s13058-014-0418-6)
Supplement: Supplementary file 2 — Additional file 2: Table S2.: 89 NCI-DTP Approved Oncology Library screen of luminobasal cells. (PDF 36 KB) [file 13058_2014_418_MOESM2_ESM.pdf]

Additional File 2: Table S2. 89 NCI-DTP Approved Oncology Library Screen of Luminobasal Cells

Drug Name	% LB ($-E_2$)	% LB ($+E_2$)	Drug Name	% LB ($-E_2$)	% LB ($+E_2$)
Acrichine	42.94	41.59	Ixabepilone	26.17	20.80
Adriamycin	29.79	28.71	Lapatinib	46.21	41.53
Allopurinol	46.30	43.94	Lenalidomide	46.44	38.83
Altretamine	43.69	44.91	Letrozole	47.72	42.97
Amifostine	45.22	40.20	Lomustine	46.14	44.19
Aminolevulinic acid	45.86	42.75	Megestrol acetate	44.94	37.22
Anastrozole	45.41	44.15	Melphalan	40.57	35.72
Arsenic trioxide	42.88	40.80	Mercaptopurine	50.92	44.03
Azacitidine	49.27	44.39	Methotrexate	37.63	33.79
Bleomycin	35.81	31.52	Methoxsalen	43.55	43.85
Bortezomib	45.71	7.00	Mithramycin	5.28	6.97
Busulfan	44.90	39.17	Mitomycin C	40.59	40.18
Capecitabine	45.55	37.25	Mitotane	43.18	40.89
Carboplatin	44.06	40.30	Mitoxantrone	34.07	38.28
Carmustine	44.17	43.10	Nelarabine	41.43	37.44
Celecoxib	45.92	40.94	Nilotinib	50.90	51.15
Chlorambucil	43.24	42.85	Nitrogen mustard	40.34	35.27
Cisplatin	44.05	42.87	Oxaliplatin	40.38	42.55
Cladribine	38.01	36.31	paclitaxel	34.26	29.92
Clofarabine	37.73	35.55	Pemetrexed	37.42	37.01
Cyclophosphamide	43.22	46.10	Pentostatin	44.48	36.85
Cytosine arabinoside	31.14	34.16	Pipobroman	44.80	48.46
Dacarbazine	46.36	42.39	Procarbazine	42.54	42.19
Dactinomycin	5.72	6.50	Raloxifene	44.07	39.93
Dasatinib	44.10	46.28	Rapamycin	46.98	39.63
Daunorubicin	20.56	21.92	Sorafenib	44.09	34.63
Decitabine	42.93	35.84	Streptozocin	43.36	40.66
Dexrazoxone	42.74	40.71	Sunitinib	48.18	41.85
Docetaxel	24.90	19.39	Tamoxifen	42.73	36.54
**Erlotinib**	10.25	7.10	Temozolomide	44.95	29.99
Estramustine	45.11	39.73	Teniposide	34.86	33.97
Etoposide	35.37	34.11	Thalidomide	44.66	40.83
Everolimus	47.35	43.49	Thioguanine	48.82	41.49
Exemestane	42.94	40.42	Thiotepa	40.41	34.53
Floxuridine	36.22	38.17	Topotecan	28.09	28.75
Fludarabine	44.53	39.46	Tretinoin	40.63	41.61
Fluorouracil	44.76	42.78	Triethylenemelamine	42.68	39.16
Fulvestrant	45.10	40.75	Uracil mustard	41.44	36.81
**Gefitinib**	9.24	8.98	Valrubicin	37.63	32.85
Gemcitabine	34.18	34.66	Vinblastine	42.14	50.26
Hydroxyurea	48.09	41.09	Vincristine	38.75	45.12
Ifosfamide	42.81	39.50	Vinorelbine	40.44	47.61
Imatinib	43.67	42.07	Vorinostat	47.32	38.78
Imiquimod	45.68	39.20	Zoledronic acid	46.95	44.63
Irinotecan	43.33	39.20	**Vehicle**	45.04	39.51
