# Supplementary material for: Modeling luminal breast cancer heterogeneity: combination therapy to suppress a hormone receptor-negative, cytokeratin 5-positive subpopulation in luminal disease
Source: Breast Cancer Res. 2014 Aug 13;16:418. doi: 10.1186/s13058-014-0418-6 (PMC4187339; doi:10.1186/s13058-014-0418-6)
Supplement: Supplementary file 3 — Additional file 3: Figure S1.: Luminal and luminobasal coculture assay for luminobasal-specific therapies. (PDF 2 MB) [file 13058_2014_418_MOESM3_ESM.pdf]

Additional File 3: Figure S1

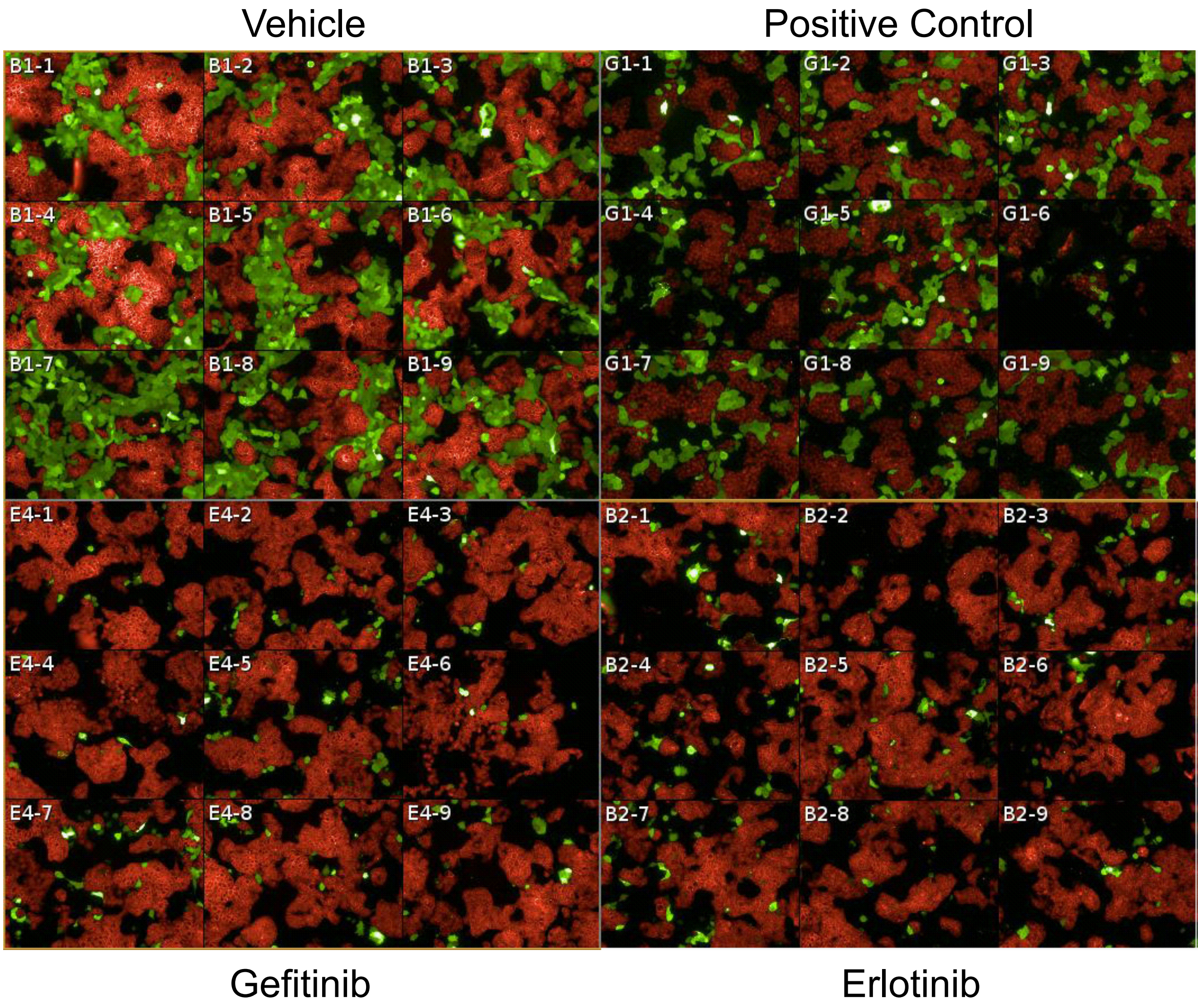

Figure S1. Luminal (Red) and Luminobasal (Green) coculture assay for luminobasal specific therapies. Top: Representative images from single wells of negative (vehicle) and positive (reduced luminobasal plating density) controls. Bottom: Representative images of wells treated with EGFR inhibitors Gefitinib and Erlotinib (1μM).
