# Supplementary material for: Modeling luminal breast cancer heterogeneity: combination therapy to suppress a hormone receptor-negative, cytokeratin 5-positive subpopulation in luminal disease
Source: Breast Cancer Res. 2014 Aug 13;16:418. doi: 10.1186/s13058-014-0418-6 (PMC4187339; doi:10.1186/s13058-014-0418-6)
Supplement: Supplementary file 4 — Additional file 4: Figure S2.: Volcano plot of differentially expressed genes in pure luminal (pLUM) versus pure luminobasal (pLB) cells. (PDF 611 KB) [file 13058_2014_418_MOESM4_ESM.pdf]

Additional File 4: Figure S2

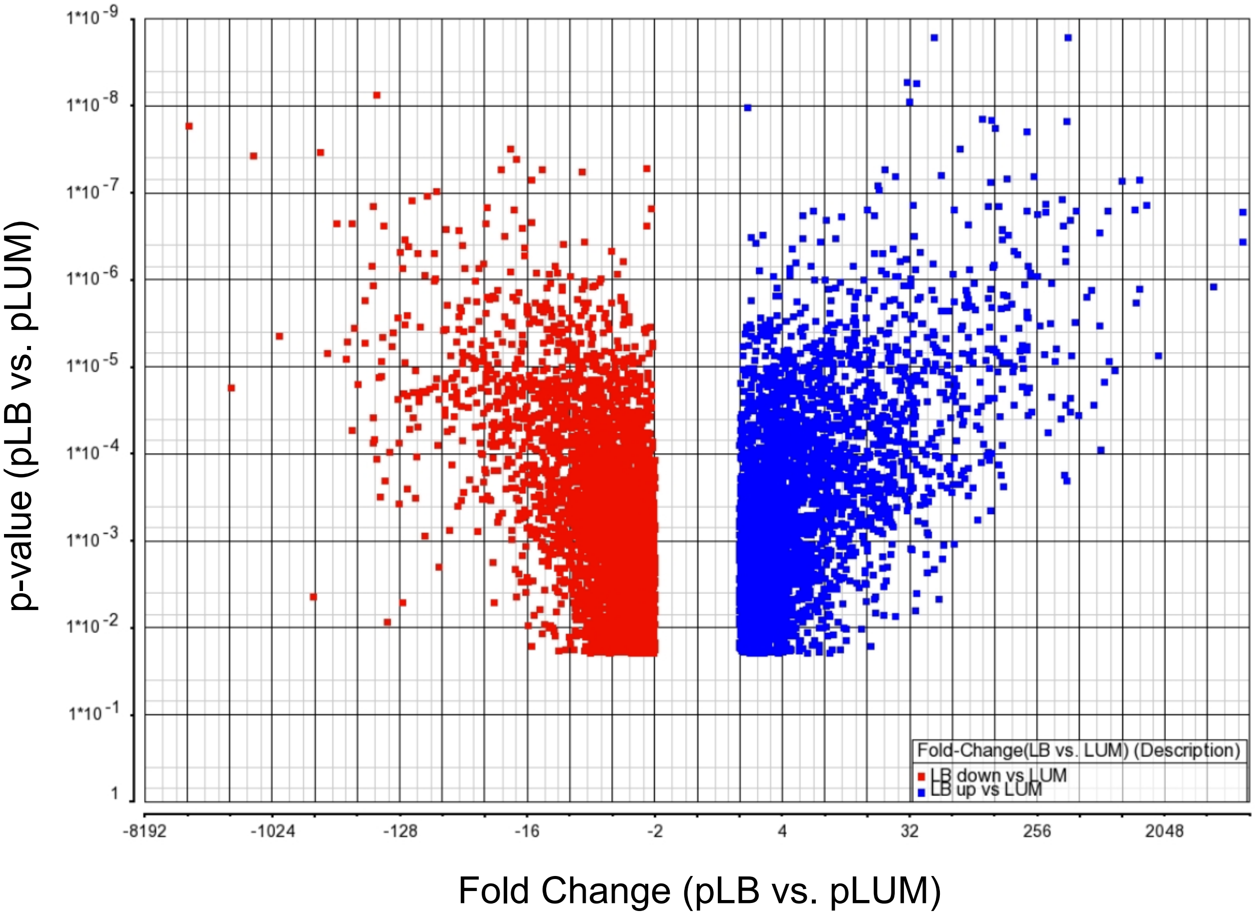


Figure S2. Volcano plot of differentially expressed genes (fold change - pLB vs. pLUM) versus the p-values (pLB vs. pLUM). Red dots: 3,970 dowregulated genes (pLB down vs. pLUM). Blue dots: 3,676 genes upregulated (pLB up vs. pLUM). Genes with a 2 fold experssion difference and cutoff p-value < 0.05 were defined as significant.
