# Supplementary material for: Modeling luminal breast cancer heterogeneity: combination therapy to suppress a hormone receptor-negative, cytokeratin 5-positive subpopulation in luminal disease
Source: Breast Cancer Res. 2014 Aug 13;16:418. doi: 10.1186/s13058-014-0418-6 (PMC4187339; doi:10.1186/s13058-014-0418-6)
Supplement: Supplementary file 5 — Additional file 5: Figure S3.: Epidermal growth factor receptor (EGFR) expression in luminal versus luminobasal subpopulations: fluorescence-activated cell sorting (FACS). (PDF 404 KB) [file 13058_2014_418_MOESM5_ESM.pdf]

Additional File 5. Figure S3

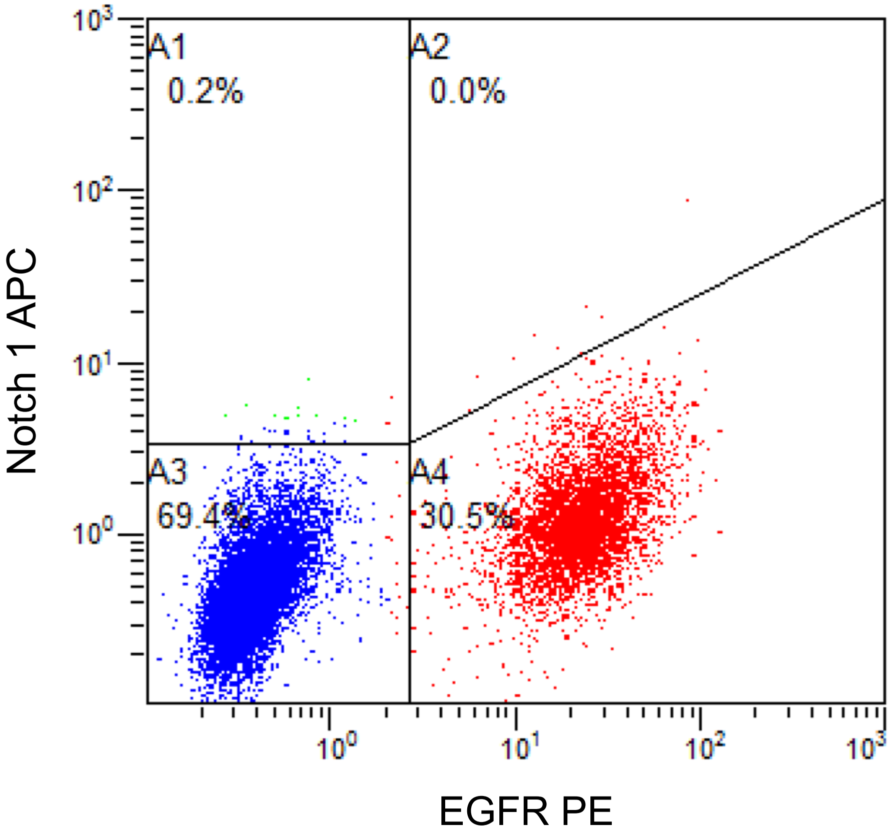

Figure S3. FACS sorting of Luminal (pLUM) and Luminobasal (pLB) subpopulations. pLUM and pLB cells were taken from culture and mixed in equal ratio, then sorted with Epidermal Growth Factor Receptor (EGFR-PE; x-axis) and Notch1-APC (y-axis). pLB cell population (red) is positive for EGFR, while pLUM cell popluation (blue) is EGFR negative.
