# Supplementary material for: Modeling luminal breast cancer heterogeneity: combination therapy to suppress a hormone receptor-negative, cytokeratin 5-positive subpopulation in luminal disease
Source: Breast Cancer Res. 2014 Aug 13;16:418. doi: 10.1186/s13058-014-0418-6 (PMC4187339; doi:10.1186/s13058-014-0418-6)
Supplement: Supplementary file 6 — Additional file 6: Figure S4.: Combined targeting of luminal and luminobasal cells: Antiestrogen/Gefitinib treatment of mixed-cell tumor models. (PDF 6 MB) [file 13058_2014_418_MOESM6_ESM.pdf]

Additional File 6. Figure S4A

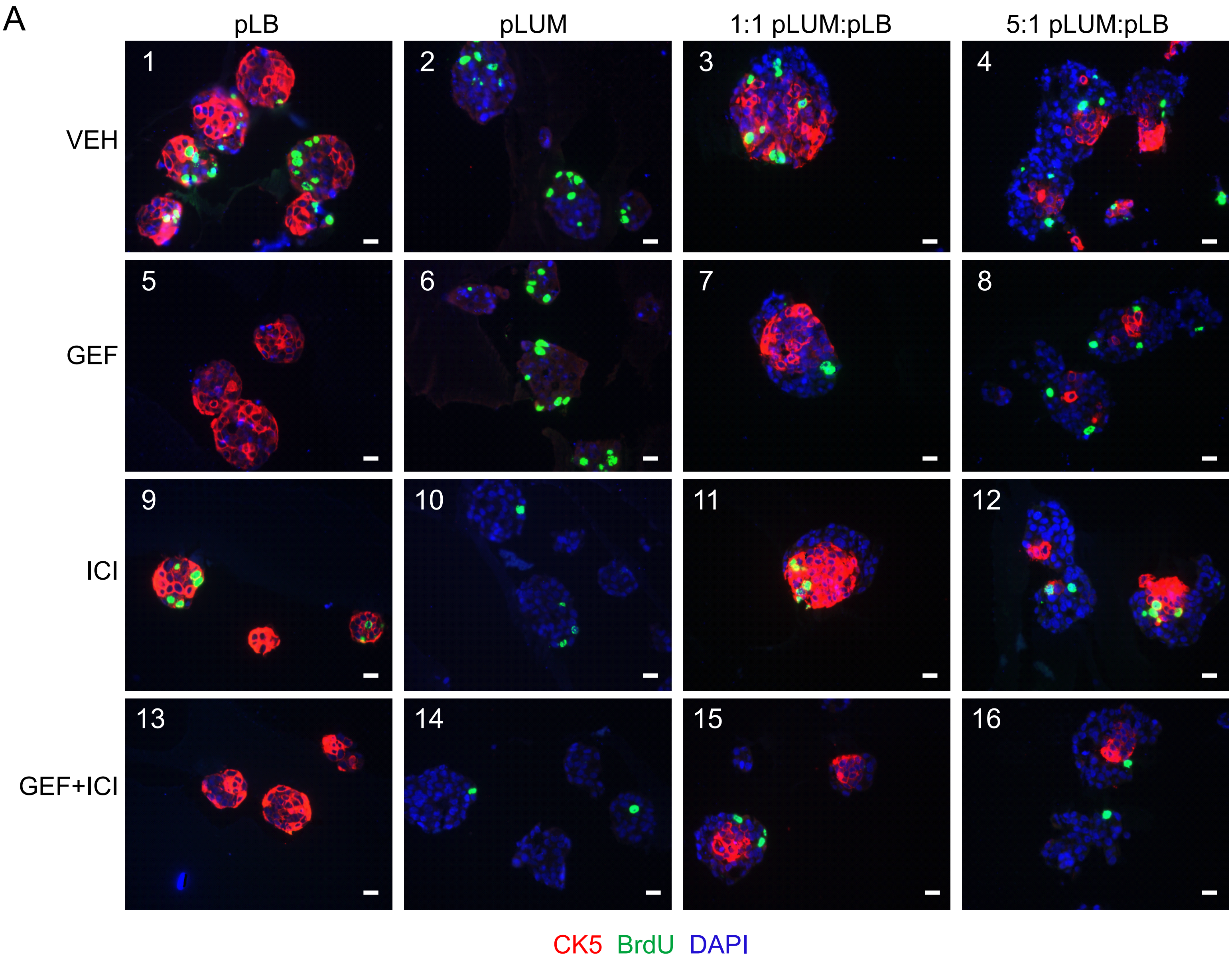

Additonal File 6. Figure S4B

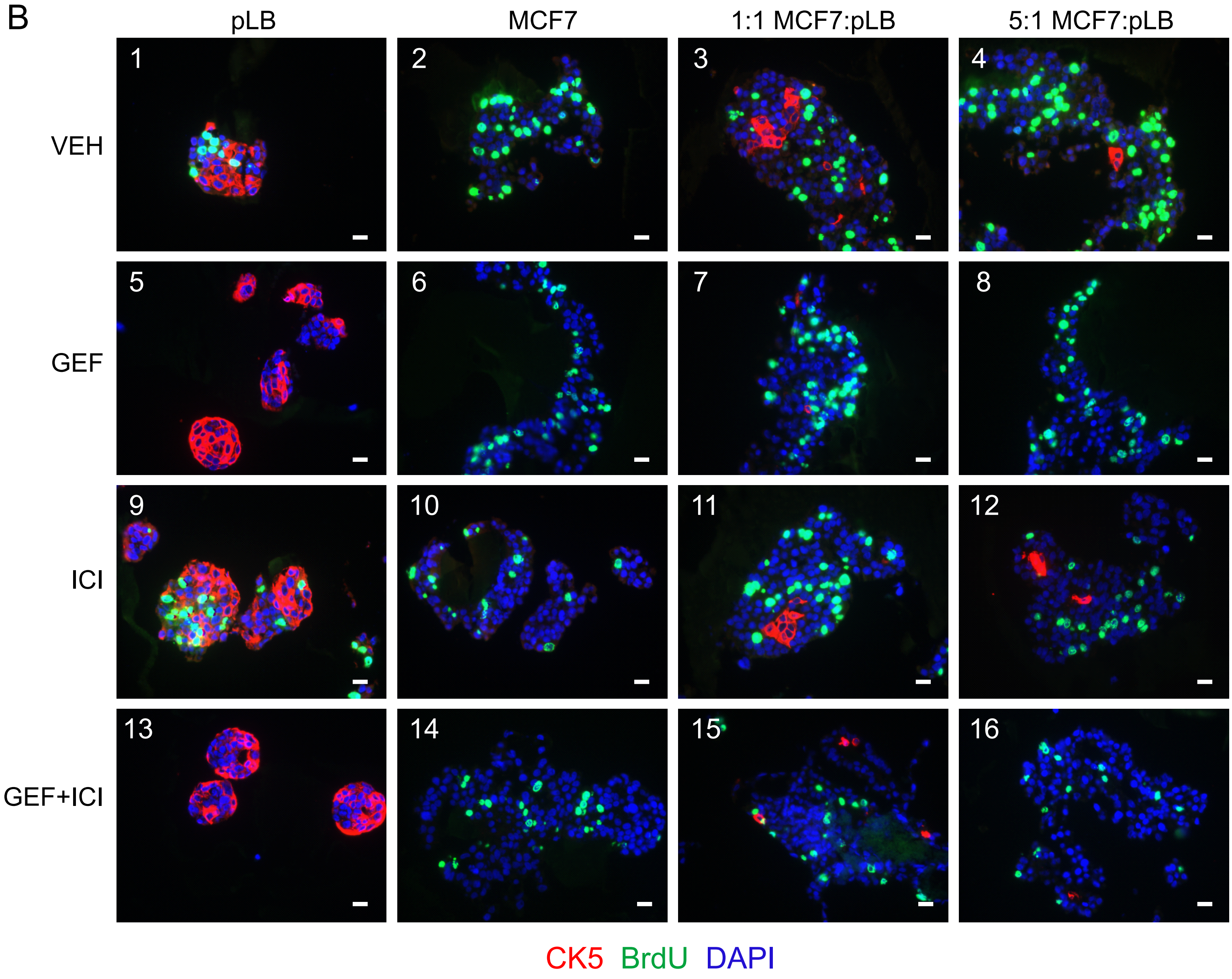

Figure S4. Combined targeting of luminal and luminobasal cells with antiestrogen/gefitinib treatment of mixed-cell tumor models. Representative images of 3D tumor models (**A**; LUM:LB, **B**; MCF7:LB) under control, ICI and/or Gefitinib treatment. Scale bars equal 20μM.
