# Supplementary figures and images for: Modeling luminal breast cancer heterogeneity: combination therapy to suppress a hormone receptor-negative, cytokeratin 5-positive subpopulation in luminal disease
Source: Breast Cancer Res. 2014 Aug 13;16:418. doi: 10.1186/s13058-014-0418-6 (PMC4187339; doi:10.1186/s13058-014-0418-6)

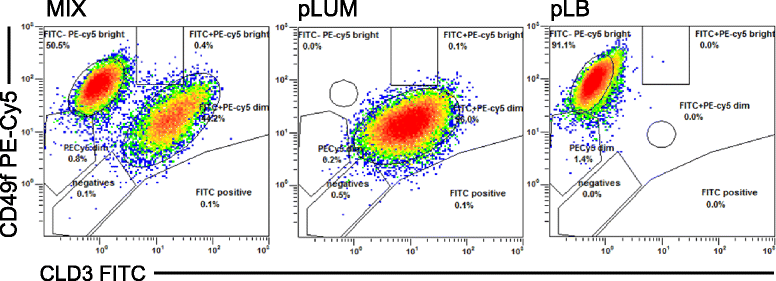

Supplement: Supplementary file 7 — Authors’ original file for figure 1 [file 13058_2014_418_MOESM7_ESM.gif]

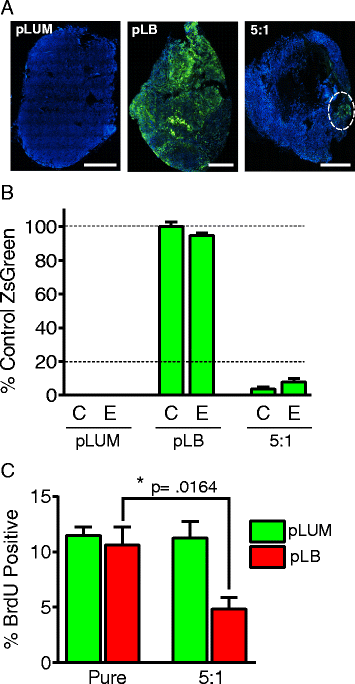

Supplement: Supplementary file 8 — Authors’ original file for figure 2 [file 13058_2014_418_MOESM8_ESM.gif]

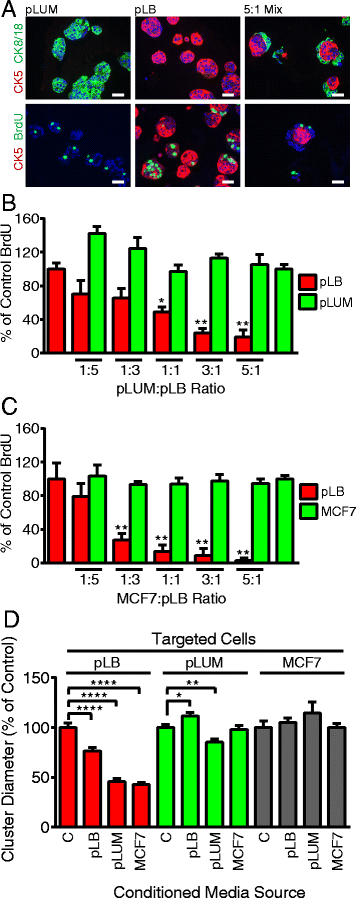

Supplement: Supplementary file 9 — Authors’ original file for figure 3 [file 13058_2014_418_MOESM9_ESM.gif]

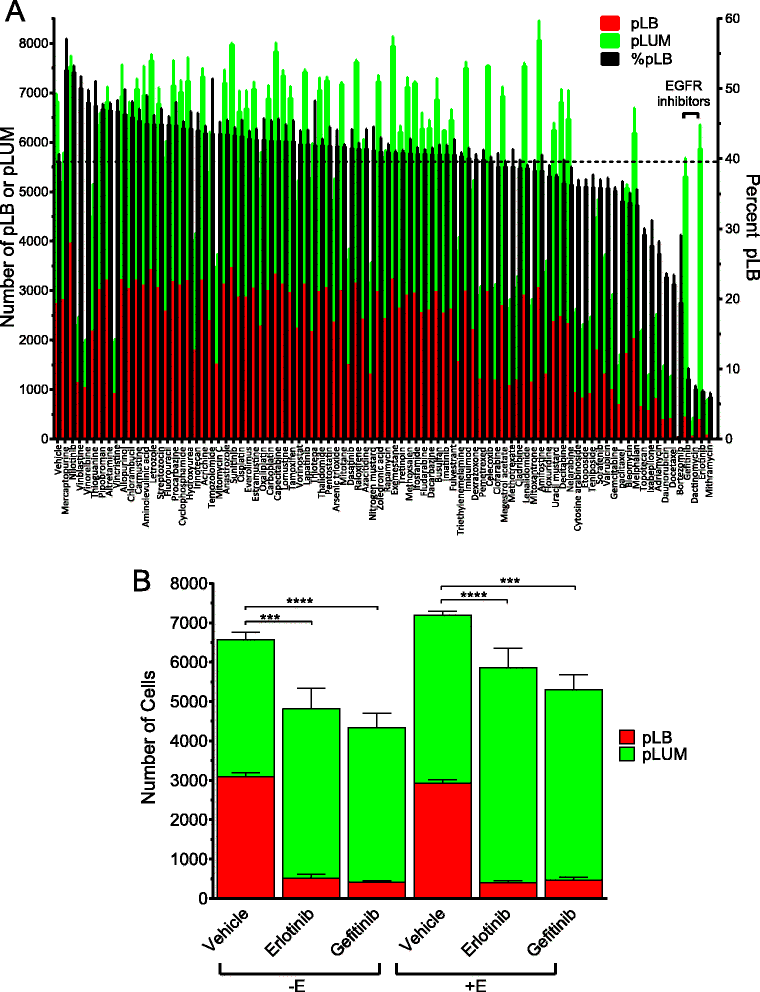

Supplement: Supplementary file 10 — Authors’ original file for figure 4 [file 13058_2014_418_MOESM10_ESM.gif]

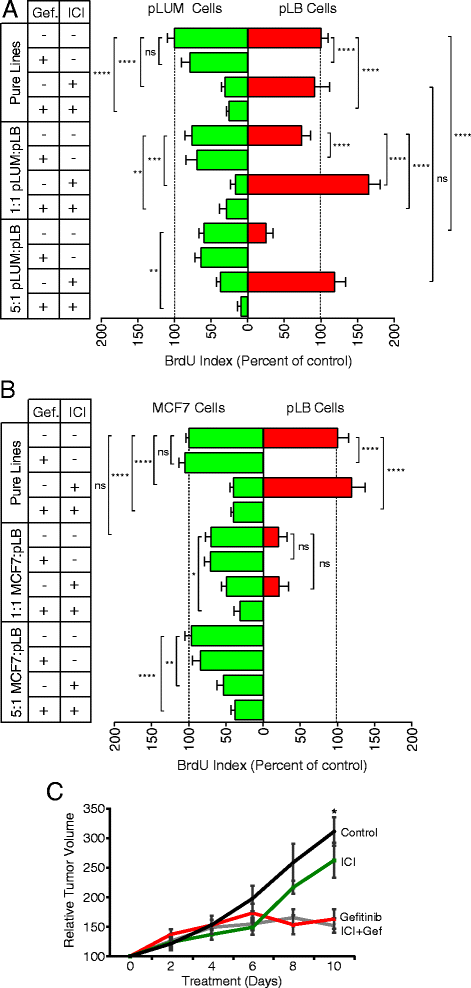

Supplement: Supplementary file 11 — Authors’ original file for figure 5 [file 13058_2014_418_MOESM11_ESM.gif]
